# Systemic delivery of TLR9-activating/STAT3-blocking oligonucleotides induces leukemia regression

**DOI:** 10.1186/2051-1426-2-S3-P107

**Published:** 2014-11-06

**Authors:** Qifang Zhang, Dewan Md Sakib Hossain, Sergey Nechaev, Ralf Buettner, Piotr Swiderski, Agnieszka Jozwiak, Stephen J Forman, Ravi Bhatia, Ya-Huei Kuo, Marcin Kortylewski

**Affiliations:** 1 City of Hope Cancer Center, Duarte, Duarte, CA, USA

## 

**Figure 1:**
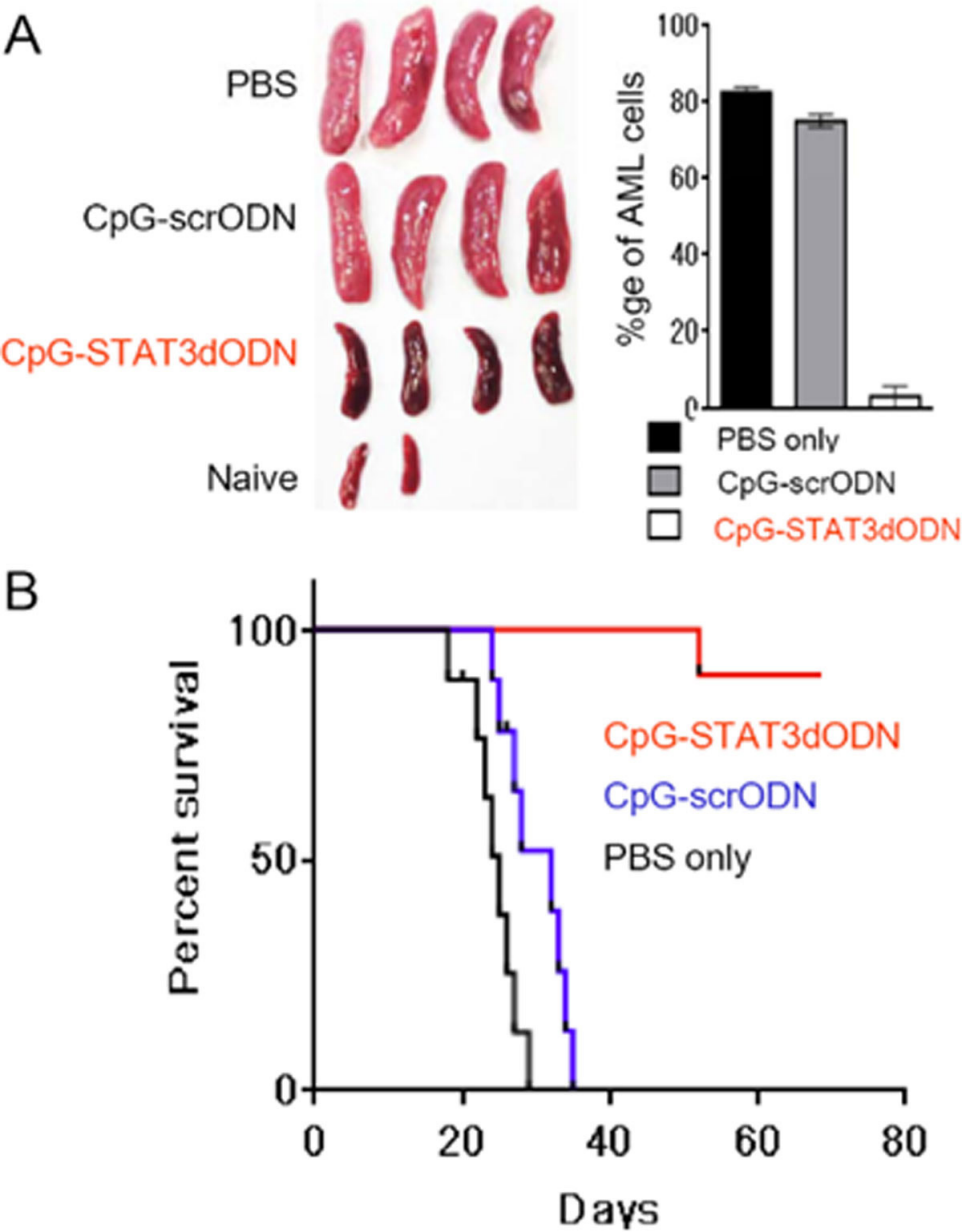


Inhibition of transcription factors (TF) that drive tumor progression and immune evasion, such as STAT3, remains a challenge for pharmacological drugs. Blocking STAT3 binding to DNA using specific decoy oligodeoxynucleotides (dODN) provides an alternative targeted inhibitory strategy. To achieve STAT3 inhibition specifically in antigen-presenting cells, we linked STAT3dODN to a TLR9 ligand, CpG ODN as successfully done before for delivery of siRNA molecules [1,2]. The CpG-STAT3dODN conjugates are quickly internalized by both human and mouse TLR9-positive target cells, such as dendritic cells (DCs), macrophages and B lymphocytes as well as by myeloid leukemia and B lymphoma cells. In contrast, the uptake of unconjugated decoy molecules by immune cells was minimal as expected. After uptake, CpG-STAT3dODN molecules bind and sequester activated STAT3 proteins, thereby inhibiting their transcriptional activity as verified using confocal microscopy, gel retardation and reporter gene assays. The hairpin design and partial phosphorothioation of the backbone increases half-life of the CpG-STAT3dODN in human serum to over 48 h. Therefore, we assessed the feasibility of using CpG-STAT3dODNs for systemic administration against disseminated human TLR9+ acute myeloid leukemia (AML). As shown in xenotransplanted MV4-11 AML model, repeated daily intravenous injections of CpG-STAT3dODN (5 mg/kg) resulted in potent and direct antitumor effects. CpG-STAT3dODN, but not control conjugates, effectively eliminated leukemic cells from all tested organs including bone marrow within two weeks from the initiation of the study. The antitumor efficacy of this strategy is greatly enhanced in immunocompetent mice. Systemic administration of CpG-STAT3dODN induced regression of the syngeneic mouse *Cbfb/MYH11 *leukemia from blood, spleen and bone marrow within 12 days of treatment (Figure 1A). The two pronged, cytotoxic and immunostimulatory effects of this strategy resulted in long-term survival of the majority of mice (Figure 1B). The potent immune activation was associated with enhanced expression of antigen-presenting and co-stimulatory molecules not only on the surface of DCs but also on differentiating AML cells. These immunostimulatory effects correlated with activation and infiltration of CD8+ T cells into various organs with concomitant reduction in regulatory T cell numbers. Our findings highlight the potential of using CpG-STAT3dODN for the two-pronged TLR9/STAT3-targeted immunotherapy of human AML and potentially other TLR9-positive blood cancers.

*This project described was supported by the National Cancer Institute of the National Institutes of Health under award number R01CA155367 to M.K*.
